# Development and Effectiveness Evaluation of 360-Degree Virtual Reality-Based Educational Intervention for Adult Patients Undergoing Colonoscopy

**DOI:** 10.3390/healthcare12141448

**Published:** 2024-07-20

**Authors:** Minju Gwag, Jaeyong Yoo

**Affiliations:** 1 Department of Nursing, College of Health and Welfare and Education, Gwangju University, Gwangju 61743, Republic of Korea; gwakmj@gwangju.ac.kr; 2 Department of Nursing, College of Medicine, Chosun University, Gwangju 61452, Republic of Korea

**Keywords:** colorectal cancer (CRC), colonoscopy screening, bowel preparation, virtual reality (VR), educational intervention, anxiety reduction, patient compliance

## Abstract

Providing patients with accurate and organized information about colonoscopy, while reducing anxiety, is critical to the procedure’s success. This study evaluated the impact of an immersive 360° virtual reality (VR)-based educational intervention for first-time adult colonoscopy patients regarding anxiety, attitudes, knowledge, compliance with bowel preparation, and bowel cleanliness. A quasi-experimental design with a non-equivalent control group and non-synchronized pretest–post-test clinical trial was conducted with 40 patients in the experimental group and 40 in the control group. The 360° VR intervention included two sessions: precautions before colonoscopy and the colonoscopy process. The control group received education through individual verbal explanations with written materials. The findings indicated that the VR intervention significantly improved patients’ colonoscopy-related anxiety, attitudes, adherence to bowel preparation instructions, and bowel cleanliness. Utilizing 360° VR as an educational tool has the potential to enhance the effectiveness of educational programs by providing realistic information and engaging patients. These findings suggest that 360° VR has the capacity to enhance screening rates and clinical outcomes by reducing negative perceptions associated with colonoscopy. Furthermore, the application of this method can extend to diverse diagnostic testing-related nursing situations in clinical settings.

## 1. Introduction

Colorectal cancer (CRC), a malignant tumor that develops in the colon or rectum, is the third most common cancer and the second leading cause of cancer-related deaths worldwide [1]. According to GLOBOCAN, an analysis by the International Agency for Research on Cancer (IARC) that examines the incidence and mortality of 36 cancers in 185 countries, CRC was estimated to account for 1,931,590 new cases in 2020 [2]. According to the World Health Organization (WHO), the number of new cases per year is expected to exceed 3.2 million by 2040, resulting in 1.6 million deaths per year [3]. The incidence of CRC is increasing in Asian countries, including Korea, due to lifestyle changes such as the increased consumption of diets high in meat and fat and decreased physical activity [4]. CRC is characterized by the absence of symptoms in the early stages and the appearance of associated symptoms only when the disease has progressed significantly [5]. Therefore, screening for CRC is essential for its early detection and timely treatment.

Guidelines for CRC screening generally recommend annual fecal immunochemical tests (FITs) for individuals aged 50 and above [6] or those in high-risk groups and colonoscopy for those with abnormal FITs [7]. However, FITs have limited accuracy in diagnosing polyps or CRC because they are based on the detection of blood in feces [7]. Colonoscopy is considered the gold standard for the accurate diagnosis and treatment of early CRC [7,8]. Colonoscopy is a screening method that enables direct observation of the mucous membrane of the large intestine to detect precancerous lesions [5]. It allows for treatments such as polypectomy and has been reported to contribute to a reduction in the incidence and mortality of CRC [9]. In both the United States and the United Kingdom, colonoscopy is recommended every 10 years if there is no family history of colon cancer [10]. In South Korea, the recommended frequency is every 5 years [11]. A recent study reported a marked reduction in the potential risk of CRC by adjusting the age of early colonoscopy screening from 50 to 45 years [12]. This finding emphasizes the importance of colonoscopy. Although colonoscopy is effective for the early detection and treatment of CRC, screening rates are only 61% and 40.4% among adults aged 45 years and older in the United States [7] and South Korea [13], respectively.

Patients who need a colonoscopy often understand its importance but may lack clarity about the procedure and bowel preparation or experience anxiety about undergoing it [14]. The process of bowel preparation for colonoscopy is often perceived as complicated, and emotional discomfort, such as pain during the procedure or worry about being diagnosed with CRC, has been reported as a major factor contributing to lower rates of colonoscopy [7,15]. Patients who undergo their first colonoscopy may experience negative emotions in an unfamiliar hospital environment if they lack sufficient knowledge and understanding of the procedure [16]. Poor bowel preparation for colonoscopy is associated with inadequate understanding of the process and negative emotions. This can lead to the low accuracy and quality of the examination, as well as patient dissatisfaction [15,17]. Providing patients with accurate and organized information about the procedure and reducing emotional discomfort, such as anxiety, are critical to the success of colonoscopy.

Providing patients with practical information about bowel preparation for colonoscopy and ensuring that they understand the procedure are the important responsibilities of nurses [15,18]. Compared with other diagnostic tests, colonoscopy requires patients to complete several tasks at home before the procedure, either independently or with the help of family members. Therefore, it is recommended that pre-colonoscopy education and management interventions be implemented to ensure that patients are well informed about bowel cleansing and that they actively participate in colonoscopy [14,17]. However, educating patients to ensure that they understand the instructions is difficult in a real-world clinical setting owing to time and resource constraints. Consequently, outpatient clinics and colonoscopy centers primarily use printed paper documents to provide verbal explanations or mail documents to patients’ homes and provide guidance over the phone [19]. Several previous studies have reported the development and use of various audiovisual materials such as pictures, cartoons, video clips, online websites, and smartphone applications for colonoscopy patient education [15,17,19]. Recent systematic reviews and meta-analysis studies have shown that educational interventions based on pictures or videos have been shown to be more effective than those based on verbal instructions, but they are limited by the lack of vividness of the information and the difficulty in conveying sufficient information about the real-life experience of a colonoscopy [15,20]. Conventional methods of patient education are limited because patients are passive recipients of information, leading to low engagement [15]. Therefore, new and innovative educational interventions are needed to maximize patient understanding of colonoscopy beyond fragmented educational approaches such as written instructions and knowledge-based videos.

In the last few years, virtual reality (VR), which uses information technology, has been widely used in educational interventions for medical personnel and patients in the healthcare field [21,22,23,24]. VR technology immerses learners in computer-generated reality through audio and visual responses via a head-mounted display, such as a special headset or goggles [25]. Immersive VR technology is currently used for skill training of healthcare providers, cognitive and perceptual training of patients, psychological interventions, and rehabilitation [26]. Previous studies have used VR as an intervention to reduce pain and discomfort in various patient populations, including patients with cancer undergoing chemotherapy and lumbar puncture, patients with burns receiving dressings, injured patients receiving wound care, patients with neurological disorders undergoing rehabilitation, and older patients with dementia seeking cognitive improvement [23,27,28,29]. Many medical institutions in Korea use VR technology as an intervention program to reduce anxiety prior to various types of diagnostic tests such as radiography or magnetic resonance imaging scans [30,31].

VR technology, particularly fully immersive 360-degree VR, has been recognized as a valuable educational tool [21,23,32]. Compared with traditional 3D-based VR, 360-degree VR allows patients to view real-world scenes from a first-person perspective through a video and freely move their point of view in all directions [32]. It creates an experiential learning experience by providing patients with a high level of realism and immersion. It has been shown to be effective in helping individuals with the understanding of complex subjects and procedures through the enhancement of their engagement with other educational interventions [17,22,33]. Interventions that provide a full view of an actual examination, such as the preparation for colonoscopy, are more realistic and immersive than those that involve observing a 3D avatar. The use of a 360-degree VR educational intervention program for colonoscopy can effectively provide accurate knowledge by reducing the variability in information acquisition among patients, which is a limitation of traditional written and verbal education. In addition, it can reduce negative perceptions of colonoscopy, leading to increased screening rates and improved screening quality, ultimately contributing to the early detection and timely treatment of CRC. Previous studies on 360-degree VR in colonoscopy patients have primarily focused on interventions that show natural scenes accompanied by relaxing music to reduce discomfort such as pain or anxiety [16,34,35]. However, few studies have used 360-degree VR to deliver educational content on colonoscopy preparation and procedures.

Therefore, this study was conducted to develop and implement an immersive 360-degree VR-based educational intervention program for adult patients undergoing colonoscopy for the first time. The program included educational content about pre-colonoscopy preparation, the examination room environment, and colonoscopy procedures. This study aimed to evaluate the impact of a program on colonoscopy-related anxiety, attitudes, knowledge, and compliance with instructions related to bowel preparation and cleanliness. The following hypotheses were examined in this study: First, colonoscopy-related anxiety will show a greater decrease in the VR intervention group than in the control group. Second, the attitudes toward colonoscopy will show more positivity in the VR intervention group than in the control group. Third, knowledge of bowel preparation instructions will show a greater improvement in the VR intervention group than in the control group. Fourth, compliance with the guidelines for bowel preparation will be higher in the VR intervention group than in the control group. Fifth, bowel cleanliness during colonoscopy will be better in the VR intervention group than in the control group.

## 2. Materials and Methods

### 2.1. Study Design

This study employed a quasi-experimental design with a non-equivalent control group and a non-synchronized pretest–post-test design. Given that the experimental treatment was a VR educational intervention conducted in an outpatient setting, there was a concern that contamination might have occurred due to the spread between the experimental and control groups. To address this issue, the intervention and data collection for the control group were conducted first and then applied to the experimental group after a time difference.

### 2.2. Study Participants and Setting

The subjects of this study were adults who underwent colonoscopy for the first time at a specialized endoscopy center in City G, Korea. The subjects were sampled conveniently. The inclusion criteria were as follows: (a) adults aged 19–65 years and (b) undergoing colonoscopy for the first time. Those who had no cognitive problems, could read and write Korean, and had no difficulty understanding or participating in the study were selected. Only subjects who signed a written informed consent form to participate in the study were included. Those who did not agree to participate were excluded. The following subjects were excluded from this study: those currently suffering from a serious illness, those taking psychotropic medications, those suffering from severe constipation or inflammatory bowel disease, those with limited communication capabilities due to visual or hearing impairment, and those who had been advised by a physician to limit the use of head-mounted display (HMD) equipment for VR because of severe dizziness.

The required number of participants was calculated using G*Power 3.1.9.7 with 34 participants per group for a total of 68 participants. An effect size of 0.70, significance level of 0.05, power of 0.80, and allocation ratio of 1 were set for independent t-test analysis. An effect size of 0.70 is the average size observed in previous studies analyzing the efficacy of experimental trials and meta-analyses of systematic reviews of VR-based interventions in adult patients [28].

**Figure 1:**
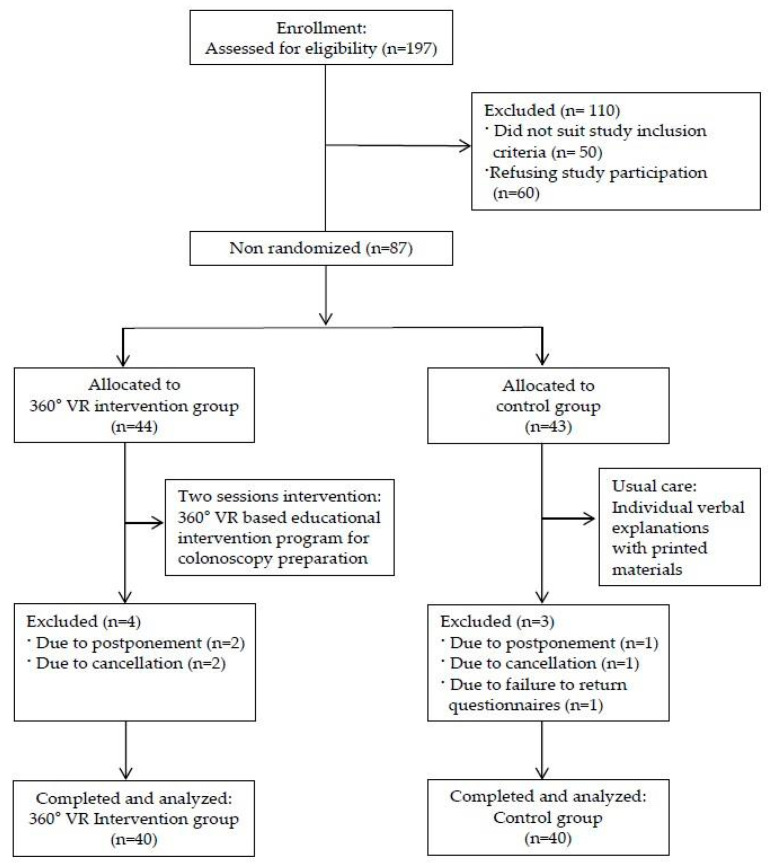
Flow diagram of the study.

Out of the initial 197 eligible subjects, 50 were excluded for not meeting the inclusion criteria, and an additional 60 declined to participate. Consequently, a total of 87 subjects were included in the study. A convenience sample of 44 participants in the experimental group and 43 participants in the control group were selected. Throughout the study, four patients from the experimental group and three from the control group were dropped from each arm due to colonoscopy postponements, cancelations, or failure to return the questionnaires. Consequently, the final analysis included 80 patients (*n* = 40 per group). (Figure 1).

### 2.3. Measurements

The main variables were self-reported using a structured questionnaire. Bowel cleanliness was assessed by a gastroenterologist who performed the colonoscopy directly and recorded the results on the case report form (CRF). The instruments used in this study were obtained from the original authors via email.

#### 2.3.1. Socio-Demographics and Clinical Characteristics

The demographic characteristics included sex, age, marital status, educational level, and economic status. The clinical characteristics included previous abdominal surgery, family history of colorectal disease, smoking habits, and alcohol consumption.

#### 2.3.2. Anxiety

Anxiety was measured using Spielberger’s State-Trait Anxiety Inventory (STAI) [36], standardized for Koreans by Kim [37], with 20 items to measure state anxiety, as the emotional state of the participants regarding the situation of colonoscopy was being examined in this study. Each item was rated on a 4-point Likert scale ranging from 1 (“not at all”) to 4 (“very much so”). The scale comprised 10 positive and 10 negative items, with the negative items reverse-scored. The scores ranged from 20 to 80, with higher scores indicating greater anxiety. The reliability of the instrument was Cronbach’s α = 0.92 in Spielberger’s study [36], 0.87 in Kim’s study [37], and 0.97 in this study.

#### 2.3.3. Attitude toward Colonoscopy

Questions regarding attitudes toward colonoscopy and preparation for colonoscopy were included in the tool developed by Amlani, Radaelli, and Bhandar [38] to determine the public’s knowledge of and attitudes toward colonoscopy. The questions were translated into Korean after being reviewed by two nursing professors. Each question was rated on a 5-point Likert scale, with 1 indicating “strongly disagree” and 5 indicating “strongly agree.” There were four positive and four negative items, with the negative items inverted. The scores ranged from 8 to 40, with higher scores indicating a more positive attitude toward colonoscopy. The reliability of the instrument in this study was Cronbach’s α = 0.66.

#### 2.3.4. Knowledge of Colonoscopy Preparation

Knowledge about taking bowel-cleansing solutions and dietary guidelines for bowel preparation were measured using an instrument developed by Yu [39], based on the guidelines of the Korean Society of Gastrointestinal Endoscopy, and modified by Cho and Kim [40]. Knowledge about taking bowel cleansers (how to take them, time, interval, storage method, etc.) consists of 5 questions, and knowledge about diet (types of foods that should be restricted, duration of diet, type of diet the day before the test, etc.) consists of 6. Each question is worth 1 point for a correct answer and 0 points for an incorrect answer. Higher scores indicate a higher level of knowledge. The dichotomous instrument reliability was based on the Kuder–Richardson formula value, which was 0.84 in Cho and Kim’s study [40] and 0.81 in this study.

#### 2.3.5. Compliance with the Guidelines for Bowel Preparation

Compliance with bowel preparation guidelines was measured using an instrument developed by Yu [39], based on the guidelines of the Korean Society of Gastrointestinal Endoscopy, and modified by Cho and Kim [40]. Compliance with bowel cleansers consists of five questions, and compliance with dietary guidelines consists of six questions. Each question is scored on a 4-point scale with a score from 4 (very well) to 1 (not at all well). Higher scores indicate higher levels of compliance. In the study of Cho and Kim [40], the reliability of the instrument showed a Cronbach’s α value of 0.80, whereas in this study, the reliability corresponded to 0.79.

#### 2.3.6. Bowel Cleanliness

The Aronchick Bowel Preparation Scale [41] was used to assess the degree of bowel cleanliness, which aims to remove fecal material while preserving the mucosa and blood vessels to ensure clear visualization of the colonic mucosa free of fecal residues. Bowel cleanliness was rated as excellent (1), good (2), fair (3), poor (4), or inadequate (5). The Aronchick Bowel Preparation Scale is a highly validated measure of bowel cleanliness. The following scores are used: “excellent” when no solid stool is observed and a small amount of clear liquid is present that is easily removed by suction; “good” when no solid stool is observed and a large amount of clear liquid is present that can be removed by suction; “fair” when a small amount of semi-solid stool is observed that is difficult to remove by washing; “poor” when solid stool is observed or the amount of semi-solid stool is large and cannot be removed by suction; and “inadequate” when the rectum is full of solid stool and cannot be examined. A gastroenterologist with more than 10 years of experience confirmed the cleanliness of the colon through direct observation during the actual colonoscopy. The degree of bowel cleanliness was assessed using the Aronchick score recorded on a CRF. The gastroenterologist was blinded to patient allocation status.

### 2.4. Research Process

#### 2.4.1. Development of VR Intervention Program

**Figure 2:**
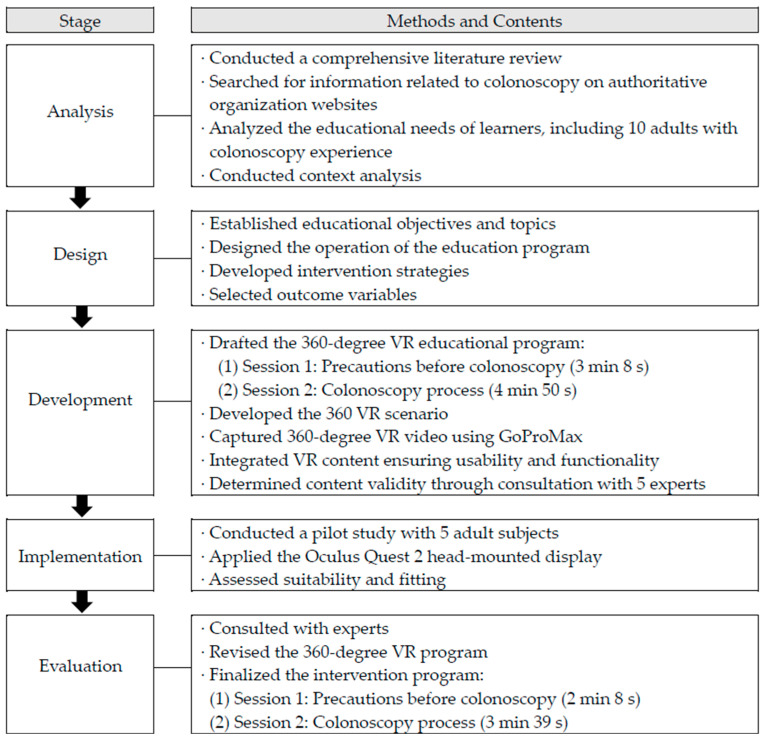
Development steps of the 360-degree VR educational intervention program.

The conceptual framework of this study is based on Lave and Wenger’s situated learning theory [42]. Situated learning is a teaching and learning model that connects theoretical and practical knowledge through a virtual experience. It assumes that “knowledge” has meaning and value when it is transmitted in the context in which it is to be applied and generalized through that context. In this study, adult patients received VR education within the context of a realistic colonoscopy scenario. The 360-degree VR educational intervention program was developed based on the ADDIE model, a pedagogical development method [43]. The program followed the processes of analysis, design, development, implementation, and evaluation (Figure 2).

(1) Analysis: A comprehensive literature review was conducted, including previous studies and searches for information related to colonoscopy on the websites of authoritative organizations to organize the content of the educational intervention in this study. In addition, to reflect the needs of the participants, the educational needs of 10 adults with colonoscopy experience were surveyed and analyzed.

(2) Design: Educational objectives, program topics, and intervention strategies were established based on a literature review and the educational needs analysis conducted in the preceding step [43]. To achieve the aforementioned objectives, it was concluded that 360-degree VR represents the optimal delivery method, maximizing the sense of presence and immersion.

(3) Development: Educational VR programs were developed based on the selected educational topics. The details of the program were constructed based on the following sources: existing educational materials and literature on colonoscopy, the latest clinical guidelines of the Korean Society of Gastroenterology [44] and the American Gastroenterological Association [5], the distribution materials of the bowel cleanser manufacturer, and the needs of patients identified in the analysis phase. The 360-degree VR educational intervention program was divided into two sessions. The primary content of Session 1 was “precautions before colonoscopy”, which was designed to enhance bowel cleansing adherence. The second session, titled “the colonoscopy process”, contained comprehensive information about the colonoscopy procedure. The objective was to reduce patient anxiety and foster a positive attitude toward colonoscopy.

The 360-degree VR video was captured using GoPro MAX (regulatory model number: SPCC1) and edited with special effects added using Adobe Premiere Pro 22.0. The VR video was designed for implementation on an Oculus Quest 2 head-mounted display (HMD) developed by Meta Inc. It was produced over a three-month period, from May 2022 to July 2022. The VR was initially developed with a duration of 3 min and 8 s in session 1 and 4 min and 50 s in session 2. The content validity of the VR was evaluated by a group of experts, including an endoscopist with over ten years of experience, a head nurse employed in a gastrointestinal endoscopy center, and three endoscopy unit nurse practitioners.

(4) Implementation: To determine the most suitable duration for the 360 VR scenario, a small pilot study was conducted with five adult subjects who were scheduled to undergo colonoscopy. The participants expressed appreciation for the opportunity to observe a colonoscopy procedure in a realistic manner and found the overall flow to be smooth and well organized. The comments indicated the need for improvement in the following areas: reducing the length of the VR video, eliminating redundancy in the content, and incorporating special effects such as subtitles.

(5) Evaluation: Based on the pilot results and expert consultation, the colonoscopy scenario and 360-degree VR video were revised and improved. The final version of the intervention program consisted of sessions 1 (2 min 8 s) and 2 (3 min 39 s). 

#### 2.4.2. Research Assistant Training

A nurse with more than 10 years of experience in the endoscopy room and in conducting interventional research was selected as the research assistant. The researcher provided comprehensive training to the research assistant regarding the purpose, methodology, and intervention protocols of the study. A standardized script was developed to guide the intervention delivery procedure, and the research assistant received extensive training during its implementation. The researcher conducted regular monitoring and verification to ensure that the standardized intervention was delivered in an accurate and consistent manner.

#### 2.4.3. Pre-Test

The pre-test was administered by trained research assistants to adult participants who provided written informed consent. The control group underwent the procedures between September 2022 and October 2022, whereas the intervention group underwent the same procedures between February 2023 and April 2023. Patients who consented to participate in this study were queried at the time of scheduling their colonoscopy regarding their anxiety, attitude, knowledge of colonoscopy preparation, socio-demographics, and clinical characteristics. The questionnaires were administered individually in a separate room within the outpatient department, with each respondent spending approximately 10–15 min completing the questionnaire. After the pre-survey, both the intervention and control groups were informed that participation in colonoscopy-related intervention programs at other institutions or in other clinical studies would be prohibited.

#### 2.4.4. Experimental Intervention

The 360-degree VR-based educational intervention program was conducted in two sessions for the intervention group from the time of scheduling the colonoscopy until the day of the examination. The first session was conducted at the time of scheduling the colonoscopy, and the second was conducted one hour before the colonoscopy on the day of the test. These two sessions were designed to address the challenges of adult patients undergoing colonoscopy attending multiple in-person training sessions.

The details of the two-session VR intervention are as follows: “precautions before colonoscopy (Session 1)” and “colonoscopy process (Session 2)”. (Table 1).

Session 1 consisted of a 2 min 8 s instructional segment on how to prepare and take a bowel cleanser (100 s), how to eat the right food (10 s), whether to take medication before the test (10 s), and the importance of bowel preparation (8 s). Session 2 consisted of 3 min 39 s of content, including awareness of preparation for colonoscopy (66 s), exploration and interaction with an actual examination room environment (33 s), information about pre-colonoscopy (11 s), procedure information (51 s), recovery after colonoscopy (28 s), and post-colonoscopy dietary guidelines and follow-up care (30 s). The first two sessions were conducted concurrently on the day of the appointment, while the second session was delivered one hour prior to the scheduled colonoscopy.

The VR intervention was conducted by a trained research assistant in a private, single-person waiting room at the endoscopy center. The room was at least 4 m × 4 m in size to ensure the safety of the participants. The Oculus Quest2 HMD, developed by Meta, was used to realize a 360-degree VR video. The participants were seated on a swivel chair with wheels to facilitate 360-degree viewing while navigating the space and wearing the HMD. To ensure stable playback of the VR video using HMDs, we stored the recorded 360-degree VR video on the Oculus developer platform and implemented wireless streaming. During the intervention, the subjects were verbally informed about the potential for VR sickness and how to address it. If they felt uncomfortable, they were informed that they could immediately remove the HMD and stop viewing the VR session. The research team arranged for the subjects to seek medical care at an internal medicine outpatient clinic or emergency room if their symptoms were severe.

The control group received colonoscopy education through individual verbal explanations utilizing written educational materials traditionally provided by endoscopy laboratories. The educational materials were provided as an 8.5” × 11” sheet of paper printed on both sides. The document, written in a font size of approximately 11–12 points, consisted of four pages. It included the following details: the definition, purpose, and method of colonoscopy; pre-colonoscopy preparation; instructions on how to take bowel cleansers; the colonoscopy process; examples of post-colonoscopy complications and prognosis; and information on payment and consent forms. The section on bowel cleansers included illustrations showing how to prepare and take the bowel cleansers. The nurse provided verbal education for approximately 7–8 min, utilizing written materials. The initial educational session was conducted on the day of the outpatient visit, during which, the date of the colonoscopy appointment was discussed. The second educational session was conducted on the day of the scheduled colonoscopy. The colonoscopy process was re-explained orally for approximately three minutes, using the same written educational material.

#### 2.4.5. Post-Test

The post-test of the participants in the intervention group was administered on the day of the colonoscopy, immediately following the delivery of the VR intervention. The control group was post-surveyed on the day of colonoscopy, immediately prior to the examination. Both groups were administered the survey in the same room as the pre-survey. Participants were requested to complete self-report questionnaires regarding their anxiety, attitudes toward colonoscopy, and knowledge of and adherence to bowel preparation and dietary guidelines. A research assistant assisted respondents in completing the questionnaire if they required assistance. The average completion time for the questionnaire was approximately 10–15 min. The Aronchick Scale was used to assess bowel cleanliness, which was recorded by the attending gastroenterologist who performed the colonoscopy. This information was subsequently entered into a CRF at the end of the colonoscopy.

### 2.5. Data Analysis

The data were analyzed using SPSS IBM 27.0 for Windows and tested at a statistical significance level of 0.05. The normality of the dependent variables was tested using the Shapiro–Wilk normality test, and all variables met the assumption of normality. The homogeneity of the intervention and control groups was tested using the χ2-test, Fisher’s exact test, and an independent *t*-test. The general characteristics of the participants were calculated as means and standard deviations, frequencies, and percentages. To assess the impact of the intervention, the pre- and post-test differences between the intervention and control groups for each variable were tested using paired *t*-tests. To assess the impact of the intervention across variables, differences between the intervention and control groups for each variable were tested using independent *t*-tests. Fisher’s exact test was used to analyze the differences in bowel cleanliness levels between the groups. The presence of cells with an expected frequency of less than five was noted, necessitating the use of Fisher’s exact test instead of the χ2 test.

### 2.6. Ethical Considerations

The study was approved by the Institutional Review Board of C University Hospital (approval number: 2-1041055-AB-N-01-2021-30) prior to data collection and intervention. To recruit participants, a notice was posted on the bulletin board in the endoscopy room. Adult patients visiting the endoscopy center were approached individually and asked whether they were willing to participate in the study. Before the commencement of the study, all subjects who expressed willingness to participate were provided with a detailed explanation of the study’s purpose and process. It was made clear to all the participants that there would be no penalty for declining to participate or withdrawing from the study at any time. Written informed consent was provided and signed by the participants, to whom their anonymity and confidentiality was explained. To prevent infection and maintain hygiene during the study, participants were required to wear disposable face masks while using VR HMDs and follow hand sanitization and hygiene protocols. During the VR intervention, no subject complained of any adverse effects such as VR sickness, other physical or mental difficulties, or abnormal symptoms.

## 3. Results

### 3.1. Homogeneity Test of General and Clinical Characteristics and Dependent Variables between the Two Groups

A total of 80 participants (*n* = 40 per group) were included in the study, with 48.7% (*n* = 39) being male and 51.3% (*n* = 41) being female. The overall mean age was 49.11 ± 11.76 years, with 23.8% (*n* = 19) aged between 50 and 60 years and 27.5% (*n* = 22) aged over 60 years. The mean age of the experimental group was 48.38 ± 12.67 years, while that of the control group was 49.85 ± 10.89 years. Among the participants, 91.3% had at least a high school diploma, 76.3% were married, and 67.5% had a stable monthly income. No significant differences were observed in age, gender, marital status, education, economic status, or clinical characteristics between the two groups. The t-test was used to assess the homogeneity of the experimental and control groups in terms of colonoscopy-related anxiety, attitude, and knowledge levels. This test satisfied a normal distribution and identified no statistically significant differences (Table 2).

### 3.2. Hypothesis Testing

#### 3.2.1. Anxiety

**Hypothesis 1.**  *Colonoscopy-related anxiety will show a greater decrease in the VR intervention group than in the control group. Testing this hypothesis revealed that the VR intervention group experienced a 19.73-point decrease in anxiety, whereas the control group experienced a 1.13-point decrease. The significant difference between the two groups (t = 6.10, p < 0.001) supports this hypothesis (Table 3).*

#### 3.2.2. Attitude toward Colonoscopy

**Hypothesis 2.**  *Attitudes toward colonoscopy will be more positive in the VR intervention group than in the control group. The test results indicated that the intervention group experienced a 3.65-point increase in attitude, while the control group experienced a 0.90-point increase. The significant difference between the two groups (t = 3.08, p = 0.003) supported this hypothesis (Table 3).*

#### 3.2.3. Knowledge of Colonoscopy Preparation

**Hypothesis 3.**  *Knowledge related to bowel preparation instructions will show a greater improvement in the VR intervention group than in the control group. The knowledge score increased by 1.50 points in the intervention group and 0.93 points in the control group. There was no significant difference between the two groups (t = 1.83, p = 0.072); thus, the hypothesis was rejected. In the subdomains, there was a statistically significant difference in knowledge of taking bowel cleansers (t = 2.10, p = 0.039) but not in knowledge of dietary guidelines (t = 0.81, p = 0.420) (Table 3).*

#### 3.2.4. Compliance with the Guidelines for Bowel Preparation

**Hypothesis 4.**  *Compliance with bowel preparation guidelines will be higher in the VR intervention group than in the control group. The hypothesis was supported, with the intervention group scoring 40.43 and the control group scoring 37.18 on compliance (t = 3.07, p = 0.003), indicating a significant difference between the two groups. In the subdomains, statistically significant differences were observed in adherence to instructions for bowel cleansers (t = 2.71, p = 0.009) and adherence to dietary instructions (t = 2.66, p = 0.010) (Table 4).*

#### 3.2.5. Bowel Cleanliness

**Figure 3:**
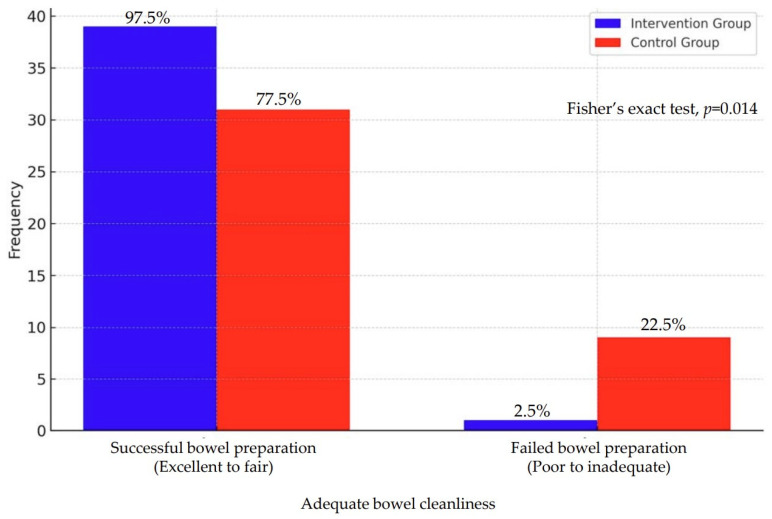
Effects of 360-degree virtual reality educational intervention program on adequate bowel cleanliness.

**Hypothesis 5.**  *Bowel cleanliness during colonoscopy will be better in the VR intervention group than in the control group. A significant difference in bowel cleanliness was observed between the intervention and control groups, as measured using the Aronchick Bowel Preparation Scale (p < 0.001). The Aronchick score was reclassified in a manner that categorized a score ranging from 1 (excellence) to 3 (fair) as a successful bowel preparation, whereas a score ranging from 4 (poor) to 5 (inadequate) was categorized as a failed bowel preparation. Therefore, this hypothesis was validated (Table 5) (Figure 3).*

## 4. Discussion

This study aimed to develop and evaluate a 360-degree VR-based educational intervention program for adult patients undergoing their first colonoscopy. The results demonstrated that the VR educational intervention had a positive effect on patients’ colonoscopy-related anxiety, attitudes, compliance with instructions related to bowel preparation, and bowel cleanliness.

Following the VR intervention, anxiety levels showed a statistically significant reduction in the experimental group compared to those in the control group. A recent rapid review of seven VR studies found that VR interventions result in a statistically significant reduction in anxiety levels [45]. A study conducted in South Korea [46] that provided 360-degree VR based on natural landscape videos, such as beaches, underwater, and spacious gardens, reported that the high-risk state anxiety group in the control group increased from 35% to 50%, whereas in the VR intervention group, it significantly decreased from 60% to 50%. Chen et al.‘s study [17], which assessed anxiety by self-rated sleep quality, also reported lower levels of pre-colonoscopy anxiety in the VR intervention group, which is consistent with the findings of this study. Furthermore, a recent review by Găină et al. [16] reported that providing VR-based imagery of natural landscapes prior to colonoscopy also reduced anxiety and nervousness associated with colonoscopy. It proposed that the provision of immersive, realistic VR imagery of the pre-colonoscopy preparation and the entire examination process may prove effective in reducing anxiety and enhancing bowel preparation. This study utilized the 360-degree VR technology to directly capture the experience of a colonoscopy in an outpatient setting. The actual procedure was filmed rather than being created in virtual space by 3D avatars to provide a more immersive experience for patients. The 360-degree VR videos were viewed from a first-person perspective on an HMD, thus affording the viewer the opportunity to experience the entire colonoscopic procedure in a realistic manner.

VR is classified as non-immersive, semi-immersive, or fully immersive based on the technical level of immersion [25]. Non-immersive VR provides a computer-generated environment; users have limited control over the physical environment. VR is typically used in video and console gaming. Semi-immersive VR provides a partial virtual environment for users by creating a sense of reality using 3D graphics. This technology is often utilized in industrial engineering fields, such as design and architecture, as well as in skill-training simulations in healthcare [23,25,26,32]. Fully immersive VR provides high-resolution content with a wide field of view through VR goggles or HMDs, maximizing the visual and sound effects for the user, enhancing immersion, and providing the highest level of realism [25]. This study utilized a fully immersive 360-degree VR experience in patients who underwent colonoscopy for the first time. The immersive environment allowed them to observe the internal space of the colonoscopy room, which enhanced their comprehension of the entire process and potentially reduced their anxiety about the procedure.

A statistically significant positive change in attitudes toward colonoscopy was observed in the experimental group compared with those in the control group. A previous study [38] examined the perceptions and attitudes toward colonoscopy among 2500 colonoscopy-naïve individuals in five European Union countries. The results indicated that 60% of the respondents reported negative attitudes toward the procedure, including feelings of anxiety, worry, and embarrassment. In particular, negative perceptions of the colonoscopy procedure were high, as were difficulties in adhering to the instructions for bowel preparation [47]. Information on colonoscopy is widely available on the Internet, including on platforms such as YouTube and social media. However, if this information is consumed without a critical evaluation of its reliability based on the latest evidence, it is likely to contain errors and misinformation [20]. The dissemination of misinformation about colonoscopy can lead to negative perceptions about this procedure among patients and the general public [38]. In addition, verbal explanations using traditional paper-based educational materials can lead to remarkable gaps in understanding, particularly in patients with low levels of education or health literacy [48]. Due to the nature of CRC and its high prevalence among older adults [1], the aforementioned gap may be even more pronounced. Furthermore, colonoscopy requires more patient preparation than other diagnostic tests, and access to the examination room may be restricted, which may lead to a lack of understanding of the test and contribute to negative attitudes [47]. The use of immersive videos that provide a 360-degree VR experience enables patients to gain a deeper understanding of bowel preparation and examination processes, which is thought to facilitate a reduction in the negative emotions commonly associated with such procedures, including anxiety and resistance.

There was no statistically significant difference in the total score of knowledge regarding bowel cleansing or dietary guidelines between the experimental and control groups. Despite the lack of 360-degree VR intervention studies, previous studies [40,49] utilizing a smartphone application for bowel preparation education and video educational interventions using a smartphone also reported positive effects on knowledge improvement. This study found that while there was partial improvement in the level of knowledge for taking bowel cleansers, this was not fully aligned with previous studies. Recent studies of educational interventions using VR in nursing have reported positive effects on performance skills, attitudes, and confidence; however, no or only limited effects were observed on knowledge levels or critical thinking [21,24]. The findings of these studies indicate that the use of less immersive VR may be more beneficial than fully immersive VR in terms of enhancing the cognitive abilities and knowledge of the subjects. This means that traditional document-based verbal explanation education and the use of colonoscopy-related photographs, cartoons, and videos had similar effects on improving colonoscopy knowledge levels. This suggests that 360-degree VR educational interventions should be used as a complementary method of education rather than as a complete replacement for traditional methods of educational delivery. Tailored experiential education using VR in addition to document-based verbal explanations may be particularly effective for older patients who are illiterate, have low health literacy, or have mild cognitive impairment.

Compliance with bowel cleansing and dietary instructions was considerably higher in the experimental group than in the control group. Previous studies [15,17,18,20] found that a VR educational intervention increased patients’ confidence in their ability to perform well and their motivation to comply by allowing them to directly experience the procedure of taking a bowel-cleansing agent compared to traditional patient education methods, including video instructions. Chen et al. [17] reported that a VR intervention increased patient adherence to instructions and ultimately improved bowel preparation outcomes. Compared to traditional educational approaches, increased patient interest and motivation to learn about and understand pre- and post-colonoscopy precautions may increase actual compliance. This supports the theoretical background of this study [42], which emphasizes that theoretical knowledge should be delivered in relation to real situations and the contexts in which it will be applied. We concluded that 360-degree fully immersive VR education helps generalize the implementation of bowel preparation instructions, ultimately leading to successful colonoscopy.

The level of bowel cleanliness showed statistically significant improvement in the experimental group compared with that in the control group. These results align with those of a previous study [17] employing VR as a pre-colonoscopy educational intervention and a study [15] that utilized various educational methods to instruct patients on key precautions, including step-by-step instructions on bowel preparation. This study utilized the actual bowel-cleansing medication that the patient would administer and created a video to explain the medication use and details of the procedure. Additionally, it compared cases of good and poor bowel cleansing and showed the impact of bowel cleansing on the examination using subtitles and explanatory screens on the VR screen, emphasizing the importance of bowel cleansing and encouraging patients to follow the instructions related to colonoscopy preparation.

The use of VR as an educational delivery method can considerably enhance the effectiveness of educational programs by providing realistic information and engaging patients [17,19,20]. VR induces high levels of bowel preparation, enhances polyp and adenoma detection rates [17], and improves colonoscopy success rates [15]. In conclusion, these findings indicate that the 360-degree VR-based colonoscopy educational intervention had a positive influence on anxiety, attitude, compliance with instructions related to bowel preparation, and bowel cleanliness in adult patients.

Despite these positive results, this study had some limitations. First, this study measured the immediate effects of the intervention but did not consider its long-term impact. Further investigation is required to ascertain whether the effects of 360-degree VR education interventions persist over time. A follow-up study with an extended intervention period and repeated measurements will allow an examination of the lasting impact of VR education. In recent times, there has been a growing emphasis on utilizing data analytics techniques, such as automated machine learning (AutoML) [50]. In Korea, a study was conducted employing deep learning algorithms on a nationwide database to facilitate the early detection and management of high-risk patients [51]. The application of AutoML to patient electronic medical record data enables the identification of patients with significant educational needs, the development of individualized educational strategies tailored to each patient, and the assessment of the effectiveness of these strategies in promoting long-term health improvements. Second, the study may not have controlled for potential exogenous variables because of convenience sampling. Future studies should be conducted using a randomized controlled experimental design to ensure rigor. Furthermore, replication of the study with a larger sample size or expansion to include individuals who have undergone colonoscopy is recommended. The sample size for this study was relatively small, with a total of 80 participants. In order to generalize the results of this study, it would be necessary to conduct the study on a larger number of subjects and to conduct it at multiple centers. A third limitation of the study is that the level of compliance with the guidelines was not directly observed but rather assessed via self-reported data from study participants. Consequently, the results of this study should be interpreted with caution, and future research should employ more objective evaluation methods, such as observational evaluation, to enhance the reliability of the findings. Fourth, this study compared a 360-degree VR intervention program with the document-based oral explanatory education provided by a single medical institution. As a result, there are limitations to analyzing the effectiveness of other educational intervention methods. Future studies should compare and analyze various intervention methods, including document-based education, verbal explanation education, demonstration education, SMS message intervention, telephone counseling, videos, and smartphone applications with 360-degree VR education interventions.

Despite these limitations, this study aimed to maximize the learning effect in adult patients undergoing colonoscopy for the first time by developing and applying a 360-degree VR educational intervention based on the ADDIE model and situational learning theory. The results of this study provide empirical support for the theoretical model, demonstrating the educational effectiveness of providing patient education in a real-world context. This approach aligns with the principles of situated learning, which emphasize immersion and focus beyond traditional document-based, explanatory patient education. Technically, this study confirmed the scalability of clinical applications of VR technology by capturing and applying 360-degree VR in nursing situations. The results indicate that this method can be effectively applied to various diagnostic test-related nursing scenarios in clinical settings. Moreover, the findings of this study may contribute to enhanced screening rates and improved clinical outcomes over time through a reduction in negative perceptions associated with colonoscopy.

## 5. Conclusions

This study presented empirical evidence supporting the effectiveness of VR-based programs in enhancing bowel preparation in adult patients undergoing colonoscopy. Additionally, the findings demonstrated the value of active VR use in clinical practice. VR educational intervention programs have the potential to be utilized as a method of nursing education, not only for colonoscopy but also for a multitude of other tests performed in clinical practice. The implementation of customized VR education programs that consider the rapidly evolving healthcare environment and specific situations of educational consumers has the potential to lead to increased nursing satisfaction and, ultimately, improved clinical outcomes by addressing patient needs. As a recommendation for future research on educational content, developing additional scenarios for various situations will prove beneficial. These scenarios could include how to respond to colonoscopy-related emergencies and how to relieve the abdominal discomfort that may occur during examinations. In addition, it is imperative to promote the development and implementation of such VR educational training. Although the accessibility of 360-degree VR filming equipment and editing programs is increasing, the financial and time resources required for VR development remain substantial. An alternative approach could be to develop standardized clinical intervention training programs in multiple languages within VR environments, spearheaded by professional societies or evidence-based guideline development organizations, and disseminate these programs to low-income countries and other regions with limited access to healthcare. With regard to technological aspects, further research is needed to develop immersive, interactive, and educational interventions that enable healthcare providers and patients to interact in real time. Such interventions should actively utilize not only virtual reality but also augmented and mixed-reality technologies to enhance learning outcomes.

## Figures and Tables

**Table 1:** Summary of 360-degree VR educational intervention program for colonoscopy.

Program Session	Components	Education Contents	Time (s)
Session 1: Precautions before colonoscopy	How to prepare and take a bowel cleanser	Visit to the hospital pharmacy Preparation and administration methods Walking exercise after intake Example of stool color check for test readiness	100
	Proper diet	Guide for dietary control using food models or photos Foods to avoid 3 days before the test	10
	Medication precautions	Consultations needed when taking medications Precautions for anticoagulants and antithrombotic drugs Advising against taking blood sugar control medications on test day Guidelines for taking high blood pressure medication on the morning of the test	10
	Importance of bowel preparation	Video explaining the complications of failed bowel preparation, such as difficulty in lesion detection during colonoscopy	8
Session 2: Colonoscopy process	Pre-colonoscopy preparation	Wearing specialized pants for colonoscopy Necessity and procedure of colonoscopy Medications to monitor during colonoscopy Securing blood vessels	66
	Exploration and interaction with an actual examination room environment	Patient monitoring devices used during the procedure Colonoscopy instruments	33
	Information about pre-colonoscopy	How to wear a nasal cannula and breathe during the test	11
	Procedure information	Positioning during the procedure Sedation methods used Simulation of sedation induction	51
	Recovery after colonoscopy	Transition from the procedure room to the recovery room Methods to reduce abdominal discomfort post-procedure	28
	Post-colonoscopy diet and follow-up care	Post-procedure diet Potential complications Outpatient visits and follow-up schedules	30

**Table 2:** Homogeneity test of general and clinical characteristics between two groups.

Characteristics	Category	Experimental Group (*n* = 40)	Control Group (*n* = 40)	χ2 or *t*	*p*
		*n* (%) or Mean ± SD			
Age (year) (average 49.11 ± 11.76)		48.38 ± 12.67	49.85 ± 10.89	0.56	0.578
Gender	Men	19 (47.5)	20 (50.0)	0.05	0.823
	Women	21 (52.5)	20 (50.0)		
Marital status	Married	27 (67.5)	34 (85.0)	5.96	0.051
	Unmarried	10 (25.0)	5 (12.5)		
	Divorce/Separation	3 (7.5)	1 (2.5)		
Education	≤Elementary school	1 (2.5)	1 (2.5)	5.98	0.179
	Middle school	3 (7.5)	2 (5.0)		
	High school	10 (25.0)	20 (50.0)		
	College/University	24 (60.0)	15 (37.5)		
	≥Graduate school	2 (5.0)	2 (5.0)		
Monthly income (average KRW 2142 K)	None	13 (32.5)	13 (32.5)	0.00	1.000
	Yes	27 (67.5)	27 (67.5)		
Participation in colorectal health education	None	37 (92.5)	35 (87.5)	0.56	0.456
	Yes	3 (7.5)	5 (12.5)		
Previous abdominal surgery	None	28 (70.0)	33 (82.5)	1.73	0.189
	Yes	12 (30.0)	7 (17.5)		
Family history of colorectal disease	None	36 (90.0)	37 (92.5)	0.16	0.692
	Yes	4 (10.0)	3 97.5)		
Presence of constipation	None	26 (65.0)	32 (80.0)	2.26	0.133
	Yes	14 (35.0)	8 (20.0)		
Current smoking	None	31 (77.5)	26 (65.0)	1.53	0.217
	Yes	9 (22.5)	14 (35.0)		
Alcohol consumption	None (non-drinking)	18 (45.0)	21 (52.5)	1.81	0.648
	1~2 times/week	17 (42.5)	15 (37.5)		
	3~4 times/week	5 (12.5)	3 (7.5)		
	7 times/week	0 (0.0)	1 (2.5)		

**Table 3:** Effects of 360-degree virtual reality educational intervention program on anxiety, attitude, and knowledge of colonoscopy preparation.

Variables	Group	Pre-Test	Post-Test	Mean Difference	T (*p* ^1^)
		Mean ± SD	Mean ± SD	Mean ± SD	
Anxiety	Exp. (*n* = 40)	54.45 ± 13.37	34.73 ± 9.37	−19.73 ± 17.16	7.27 (<0.001)
	Cont. (*n* = 40)	54.43 ± 15.03	53.30 ± 17.27	−1.13 ± 8.78	0.81 (0.422)
	T (*p* ^2^)	0.01 (0.994)	6.10 (<0.001)		
Attitude	Exp. (*n* = 40)	29.93 ± 4.76	33.58 ± 3.85	3.65 ± 4.23	−5.46 (<0.001)
	Cont. (*n* = 40)	28.73 ± 5.38	29.63 ± 5.43	0.90 ± 3.74	−1.52 (0.136)
	T (*p* ^2^)	1.06 (0.294)	3.08 (0.003)		
Knowledge of colonoscopy preparation (total score)	Exp. (*n* = 40)	9.05 ± 1.65	10.55 ± 0.55	1.50 ± 1.60	−5.90 (<0.001)
	Cont. (*n* = 40)	8.90 ± 1.72	9.83 ± 1.38	0.93 ± 1.19	−4.94 (<0.001)
	T (*p* ^2^)	0.40 (0.692)	1.83 (0.072)		
Sub 1. Knowledge of taking bowel cleanser	Exp. (*n* = 40)	3.75 ± 0.87	4.58 ± 0.55	0.83 ± 0.81	−6.92 (<0.001)
	Cont. (*n* = 40)	3.68 ± 0.92	4.13 ± 0.91	0.45 ± 0.78	−3.64 (0.001)
	T (*p* ^2^)	0.38 (0.708)	2.10 (0.039)		
Sub 2. Knowledge of dietary guidelines	Exp. (*n* = 40)	5.30 ± 1.24	5.98 ± 0.16	0.68 ± 1.25	−3.00 (0.005)
	Cont. (*n* = 40)	5.23 ± 1.27	5.70 ± 0.76	0.48 ± 0.93	−3.22 (0.003)
	T (*p* ^2^)	0.27 (0.790)	0.81 (0.420)		

Note: *p* ^1^: *p*-value by the independent sample *t*-test, *p* ^2^: *p*-value by paired *t*-test; Exp. = experimental group; Cont. = control group.

**Table 4:** Effects of 360-degree virtual reality educational intervention program on compliance with guidelines for bowel preparation.

Variables	Group	Post-Test	*t*	*p*
		Mean ± SD		
Compliance (Total)	Exp. (*n* = 40)	40.43 ± 3.25	3.07	0.003
	Cont. (*n* = 40)	37.18 ± 5.84		
Sub 1. Compliance with bowel cleansers	Exp. (*n* = 40)	18.08 ± 1.75	2.71	0.009
	Cont. (*n* = 40)	16.65 ± 2.83		
Sub 2. Compliance with dietary guidelines	Exp. (*n* = 40)	22.35 ± 2.11	2.66	0.010
	Cont. (*n* = 40)	20.53 ± 3.79		

Note: Exp. = experimental group; Cont. = control group.

**Table 5:** Effects of 360-degree virtual reality educational intervention program on bowel cleanliness.

Variables	Categories	Experimental Group (*n* = 40)	Control Group (*n* = 40)	χ2	*p* ^1^
		*n* (%)			
Bowel cleanliness by Aronchick Bowel Preparation Scale	Excellent (grading: 1)	8 (20.0)	1 (2.5)	26.567	<0.001
	Good (grading: 2)	27 (67.5)	12 (30.0)		
	Fair (grading: 3)	4 (10.0)	18 (45.0)		
	Poor (grading: 4)	1 (2.5)	8 (20.0)		
	Inadequate (grading: 5)	0 (0.0)	1 (2.5)		
Adequate bowel preparation	Successful (excellent to fair)	39 (97.5)	31 (77.5)	7.314	0.014
	Fail (poor to inadequate)	1 (2.5)	9 (22.5)		

Note: *p* ^1^: *p*-value by Fisher’s exact test.

## Data Availability

The data presented and/or analyzed during the current study are available from the corresponding author on reasonable request.
